# Influence of the Technological Process on the Biochemical Composition of Fresh Roe and Bottarga from *Liza ramada* and *Mugil cephalus*

**DOI:** 10.3390/foods9101408

**Published:** 2020-10-04

**Authors:** Francesco Corrias, Alessandro Atzei, Angelica Giglioli, Viviana Pasquini, Alessandro Cau, Piero Addis, Giorga Sarais, Alberto Angioni

**Affiliations:** 1 Department of Life and Environmental Science, Food Toxicology Unit, University of Cagliari, University Campus of Monserrato, SS 554, 09042 Cagliari, Italy; francesco.corrias@unica.it (F.C.); alessandro.atzei@unica.it (A.A.); gsarais@unica.it (G.S.); 2 Department of Life and Environmental Science, Biology Section, University of Cagliari, via Tommaso Fiorelli 2, 09126 Cagliari, Italy; giglioli.angelica@gmail.com (A.G.); vivi.pasquini@yahoo.it (V.P.); alessandrocau@unica.it (A.C.); addisp@unica.it (P.A.)

**Keywords:** bottarga, salting-out, food processing, lipid composition, squalene, metals, PCA

## Abstract

Bottarga is a high-priced delicacy with high nutritional value, and, in Italy, bottarga from mullets has been recognized to be a traditional food product. The flathead grey mullet *Mugil cephalus* and the thinlip grey mullet *Liza ramada* are the main cultured grey mullets in the Mediterranean Sea. In this study, fresh roe and bottarga from these two species were investigated to evaluate the influence of the technological process and the species on their biochemical composition and health advantages. The 1 h/200 g salting-out step did not increase the levels of NaCl in the bottarga, although it highly decreased the levels of some heavy metals like Cu and Al. Processing of fresh roe in bottarga led to an essential modification of the lipid fraction, following a general series of monousatturated fatty acid (MUFA)> poliunsutturated fatti acid (PUFA) > saturated fatty acid (SAFA) and an increase in both ω3 and ω6 in *Liza ramada*. Moreover, bottarga showed higher levels of squalene and cholesterol and an increased Essential Amino Acid/Total Amino Acid ratio (EAA/TAA) in both species. In addition to the nutritional benefits for the consumer, the process proposed in this study may represent a reliable tool for local producers to obtain a final bottarga with both a reproducible biochemical composition and organoleptic characteristics.

## 1. Introduction

Grey mullets (Mugilidae) are cosmopolitan euryhaline species that inhabit coastal, estuarine, and freshwater environments proximal to high human populations [1]. Due to their cosmopolitan distribution, they represent a popular target for commercial fisheries, reaching a whole production of 698,293 t in 103 countries in 2013 [1]. In the Mediterranean area, the main cultured grey mullet species are the thinlip *Liza ramada* (Risso, 1826) and the flathead *Mugil cephalus* (Linneo, 1758) [2]. In Italy, mullet farming is almost entirely based on extensive techniques, with coastal lagoons and semi-intensive ponds being restocked with wild juveniles [3]. *M. cephalus* is the dominant aquaculture species; it can reach a length of up to 120 cm (weight up to 5 kg) with a maximum reported age of 16 years, making it the prevalent mullet species [4], while *L. ramada* has a lower growth rate, reaching a body length of up to 70 cm (weight 2.9 kg) with a maximum reported age of 8 years [4]. Adults of *L. ramada* and *M. cephalus* live in large schools with different habits. *L. ramada* prefers surface water over sandy and dense *Posidonia oceanica* vegetation, while *M. cephalus* habitat is mostly represented by muddy bottoms and rocky seabed with vegetation. Usually, they move from the sea into brackish coastal lagoons, where they spend most of their growing phase, before migrating back to the sea to spawn [1]. Grey mullets provide several different foodstuff, including fresh fish fillets and processed roe, known as bottarga or boutargue in Europe or karasumi in Asia, for human consumption [5,6]. Bottarga is obtained from the ripening ovaries after salting and air drying. In Sardinia, salted mullet roe obtained from *M. cephalus* and *L. ramada* are considered a delicacy, equivalent to sturgeon caviar, and can reach 200 euros/kg when obtained from local Sardinian mullets. In Italy, bottarga from mullets has been recognized to be a traditional food product, and Sardinia is the main producing region, assuming an important role in the local, national, and international market [7,8,9]. *M. cephalus* fish of 1.5 to 2.5 kg produce fresh gonads ranging 300–600 g, which, after processing, yield a bottarga of 200–400 g weight and final thickness around 1.6 cm, whereas *L. ramada* fresh gonads can reach 50 g and a final bottarga of around 30 g weight and 0.8 cm thickness. The organoleptic characteristics of bottarga are related to an intense and sweet flavor, with an evident salty note but round and balanced with a slightly bitter and spicy aftertaste. Bottarga has a low level of water activity (Aw); therefore, it is not the ideal breeding ground for mold and microorganisms. The principal negative aromas in bottarga are represented by muddy and musty odors related to the presence of geosmin and 2-methylisoborneol, two compounds produced by cyanobacteria and actinomycetes, which grow in restricted and closed pounds [10]. A few papers have reported the biochemical composition of bottarga from different fishing areas [11], carried out by GC–MS and NMR techniques [12,13,14,15]. Moreover, some papers have reported the effect of the processing method on the proximate to monitor spoilage during cold storage [16] and on the rheological characteristics [17]. No paper was found in the literature reporting the influence of the processing steps on the biochemical composition of the bottarga and the composition of *L. ramada* in comparison with *M. cephalus.* The purpose of this paper is to analyze the biochemical composition and quality characteristics of ripe ovaries, fresh and after processing in bottarga, from two different grey mullet species grown in the Cabras lagoon (Sardinia, Italy). Moreover, since the two grey mullets show different behavioral attitudes, the presence of the off-flavors geosmine and 2-methylisoborneol and the concentration of some metals and metalloids were also investigated.

## 2. Materials and Methods

### 2.1. Sample Collection

Ten samples *of Mugil cephalus* and *Liza ramada* migrating ripe females were collected in 2019 (late summer) from the Cabras lagoon (39°54′46.87′′ N–8°30′33.20′′ E, Gulf of Oristano) [18]. This wetland area, one of the major brackish systems of the western Mediterranean, is traditionally devoted to fishing and aquaculture practices [19,20]. Samples were captured using low-frequency, pulsed DC electrofishing. Stunned ripe female fish of appropriate weight and size were immediately killed by spiking the brain [21], held in iceboxes, and brought to the laboratory within three hours for the processing step.

### 2.2. Processing Step

Mullets were measured for total length (TL, cm) and body weight (BW, kg). Then, the fish were dissected using a sharp stainless-steel scalpel and the ovaries were removed, avoiding the vitelline membrane breaking. The two fresh ovaries from each fish were cleaned and freed from the gonadal artery. Thereafter, they were separated, weighed, and one was used for the analysis of the fresh gonads (control sample), and the other for the processing in bottarga.

Gonad processing involves a salting-out step (1 h/200 g roes; 4% weight decrease). After that, the salt residues were removed, and gonads were placed between two wooden boards and pressed for one day (6% weight decrease). The wooden boards were previously sanitized with a solution of NaClO at 4%. After pressing, the gonads were left at atmospheric temperature (20 °C) and constant humidity (53%) and rotated each day in order to facilitate water evaporation till they reached a 30% weight loss. The dehydration step in the open air took 5–7 days. Bottarga samples were stored in vacuum containers at 5 °C till analysis. Each analysis was performed in triplicate, a total of 30 samples of fresh gonads and bottarga were analyzed in the study.

### 2.3. Chemicals

Ethylic ether, petroleum ether, hexane, methanol, and chloroform were the ultra-residue solvents of analytical grade, purchased from Merck (Darmstadt, Germany). NaCl, MgSO_4_ anhydrous and Na_2_SO_4_ anhydrous KOH and KCl were of analytical grade (Sigma Aldrich Chemie, Darmstad, Germany). Geosmin (GSM), 2-methylisoborneol (2-MIB; purity ≥95%), MSTFA, squalene, cholesterol, and dodecanone were analytical grade standards (Fluka, Sigma Aldrich Chemie, Germany). The amino acid mix solution was purchased from Merck (Darmstadt, Germany). The marine oil FAME mix analytical standard was purchased from Restek (Bellefonte, PA, USA). HNO_3_ 67–69%, H_2_O_2_ 30% (Carlo Erba, Milan, Italy), HCl 37% super pure quality (Romil Spa, Cambridge, UK) and standards of Al, As, B, Ba, Be, Cd, Co, Cr, Cu, Fe, Hg, Mn, Mo, Ni, Pb, Sb, Sn, Sr, Te, Ti, V, and Zn were of ICP grade (Carlo Erba Reagents, Milan, Italy). Double-deionized water, with a conductivity of less than 18.2 MΩ, was obtained with a Milli-Q system (Millipore, Bedford, MA, USA).

### 2.4. Moisture and Ash

Briefly, 5 g of homogenized samples (fresh gonads and bottarga) were weighed and dried at 100 °C until a constant weight was achieved (~24 h) for moisture determination. Samples were then heated to 500 °C in a porcelain crucible for five hours for carbonization and ash analysis. Moisture and ash determinations were calculated as a percentage of the fresh product.

### 2.5. Metals and Metalloids

Metals and metalloids were analyzed according to Corrias et al. (2020) [22]. Briefly, ashes obtained by carbonization were mineralized in a microwave CEM Mars6 system (CEM Italia, Bergamo, Italy). About 0.2 g of ashes was precisely weighed, and then 10 mL of a mixture of HNO_3_ and HCl (molar ratio 1:3) and 1 mL of H_2_O_2_ were added. After the mineralization program, the solutions were transferred into a 10-mL flask and brought to volume with Milli-Q water, filtered through a 0.45 μm nitrocellulose membrane filter (Whatman, Milan, Italy) in another flask and, finally, subjected to analysis. Control solvent samples were simultaneously prepared to avoid false-positives and contamination during analysis. Hg and As sample preparation was carried out using an Agilent VGA-77 (Agilent, Milan, Italy) according to Agilent application notes [23,24]. A Varian 710ES ICP Optical Emission Spectrometer (Agilent, Milan, Italy) for the simultaneous analysis of 22 metals and metalloids was used. Operating conditions: radiofrequency (RF) generator power 1.2, frequency 40 MHz; argon (99.996% purity) was used for plasma (15.5 L/min), nebulizer (200 Kpa), and optic supply (1.5 L/min). The sample uptake delay was 30 s, and the instrument stabilization delay was 15 s. Power and pressure applied were 600 W and 100 PSI for 13 min. Each measurement was made in triplicate. The limit of quantitation (LOQ) was calculated as ten times the standard deviation reading of the blank sample signal [25]. Calibration curves were calculated on five points starting from the LOQ value and were considered acceptable for *r*^2^ ≥ 0.990.

### 2.6. Analysis of the Lipid Fraction

#### 2.6.1. Total Lipid

Briefly, 1 g of homogenized sample (fresh gonads and bottarga) was accurately weighed in an extraction thimble. Lipids were extracted using the rapid extraction system for solid–liquid extraction Soxtherm (C-Gerhardt, Analytical Systems, Konigswinter, Germany) with 150 mL mixture of ethylic ether/petroleum ether (1/1), according to the AOAC method [26].

#### 2.6.2. Squalene and Cholesterol

Briefly, 0.5 g of homogenized sample (fresh gonads and bottarga) was weighed in a screw-capped vial, with 2 mL of hexane, vortex-mixed for 3 min and centrifuged for 10 min at 4000 rpm and 10 °C. The organic phase was recovered and diluted 1/10 with hexane. The analyses were carried out using a Varian CP 3800 gas chromatograph (Varian, Inc., Palo Alto, CA, USA) coupled with a Varian CP 7800 autosampler and a Varian 2000 ITMS. The column was a DB-5MS (5% phenylmethyl polysiloxane, 30 m × 0.25 mm, film thickness 0.25 μm; J&W Scientific Fisons, Folsom, CA, USA). The injector and the MS interface were set at 280 and 180 °C, respectively. The oven was programmed as follows: 60 °C (1 min) to 300 °C (3 °C/min). Helium was the carrier gas at 1 mL/min. The sample (1 μL) was injected in splitless mode (30 s). The data were processed using the Varian ChemStation. ITMS conditions were as follows: ionization voltage 70 eV, scan rate 1.6 scan/s, mass range 40–650, ionization source at 180 °C. Quantitative ions were *m/z* 386 and 69 for cholesterol and squalene, respectively. Qualifiers ions were *m/z* 353, 301, and 275 for cholesterol and *m/z* 410, 69, and 95 for squalene. A five-point calibration curve was prepared from authentic standards in the range of 1.0–100 mg/kg.

#### 2.6.3. Fatty Acid

Briefly, 5 g of homogenized samples (fresh gonads and bottarga) was accurately weighed in a screw-capped tube of 40 mL, plus 10 mL of hexane, 3 g of NaCl, and 2 g of MgSO_4_. The tube was agitated for 2 min in a vortex and for 15 min in a rotatory shaker. The tubes were then centrifuged at 4000 rpm for 10 min (10 °C temperature). Transesterification was carried out as follows: 500 µL of hexane extract plus 200 µL of alcoholic potash (KOH 2 N in MeOH) were heated and agitated in a vortex for 4 min. The organic phase was then transferred to another vial and injected in GC–MS for the analysis. Analysis was carried out using a gas chromatograph TRACE GC ULTRA and a Single Quad DSQ mass detector (Thermo Finnigan, Milan, Italy), equipped with a COMBI PAL autosampler (CTC Analytics, Zwingen, Switzerland) and a split/splitless injector. The analytical column was a Varian Factor Four VFWAX (60 m × 0.25 mm i.d. × 0.25 µm film thickness; Varian, Milan, Italy). Helium was the carrier gas at 1 mL/min. The sample of 1 µL was injected in splitless mode (1 min). The mass spectrometer detector was operated in electron ionization (EI) positive mode, with a solvent delay of 5.5 min. Injector, ion source, and transfer line temperatures were at 50, 200, and 250 °C, respectively. The oven was programmed as follows: 90 °C (1 min), raised to 160 °C (3 °C/min), then 198 °C (1 °C/min), and further to 250 °C (5 °C/min), and then held for 15 min. Peak identification was made, comparing full mass spectra (50–550 *m/z*) and retention times (r.t.) from authentic standards and the NIST MS spectra library.

### 2.7. Analysis of Protein Fraction

#### 2.7.1. Total Protein

Total protein was analyzed according to the Kjeldahl method. Briefly, 0.5 g of well-homogenized sample was weighed in the Speed-Digester flasks; 20 mL of concentrated H_2_SO_4_ (96%), 0.5 g of sodium sulfate, and a tip of copper sulfate were added to the mineralization flask. After mineralization, the solution was left to cool, and 50 mL of H_2_O MilliQ were added. When the solution reached a light blue color, the flask was inserted into the distillation unit, and a concentrated solution of NaOH was added directly to the distiller and subjected to distillation until a brownish-black color was obtained. Then, 100–150 mL were collected, and a known quantity of H_2_SO_4_ 0.5 N plus few drops of methyl red were added. After distillation, quantitative analysis was made by acid-base titration using 0.5 N NaOH.

% protein = ((a − b) * c * 100 * K)/g sample

a: mL of 0.5 N H_2_SO_4_ added to the collection flask; b: mL of titrant used (NaOH 0.5 N); c: conversion factor ml of H_2_SO_4_ 0.5 N in g of nitrogen (0.007); K: general nitrogen–protein conversion factor (6.25).

#### 2.7.2. Amino Acid Analysis

Briefly, 100 µg of homogenized samples (fresh gonads and bottarga) was weighed in an Eppendorf tube, and 250 µL of MeOH and 125 µL of CHCl_3_ were added. The tube was stored for 1 h in the dark and shaken in a vortex every 15 min. After adding 380 µL of CHCl_3_ and 90 µL of KCl 0.2 M, the tube was shaken for 3 min in a vortex and centrifuged at 14,000 rpm and 4 °C for 10 min. The hydrophilic phase was transferred in another vial and dried under a gentle nitrogen stream. For derivatization, 50 µL of methoxamine pyridine solution (104 mg/L) was added in the vial, and the solution was left in the dark for 17 h. After that, 100 µL of MSTFA was added, followed by 3 min in a vortex and 1 h in the dark. Before gas-chromatography analysis, 600 µL of dodecanone solution (20 mg/L) in hexane (IS) was added. Samples were injected (1 μL) in splitless mode into a 6850 gas-chromatograph coupled with a 5973 mass-spectrometer (Agilent Technologies, Santa Clara, CA, USA). The injector temperature was 200 °C, and the gas flow rate was 1 mL/min. The column was a DB5-MS capillary fused silica column (0.25 μm, 30 m × 0.25 mm ID; J&W Scientific, Folsom, CA, USA). The temperature program was T = 0 50 °C (10 min), then increased to 300 °C at 10 °C/min and held for 10 min. Ionization was at 70 eV in EI+, over *m/z* 50–550 mass range. Identification of free amino acids was performed using the standard amino acid mixture (alanine, arginine, aspartic acid, glutamic acid, glycine, histidine, isoleucine, leucine, lysine, methionine, phenylalanine, proline, serine, threonine, tyrosine, valine, and cystine) and the NIST08 mass spectra library.

### 2.8. Geosmine and 2-Methylisoborneol

Analyses were carried out according to Angioni et al. [10], briefly: 5 g of homogenized samples were weighed in a screw-capped tube with 3 g of NaCl, 10 mL of hexane, and 2 g of MgSO_4_ anhydrous. The tube was stirred in a vortex (2 min), and rotary shaker (15 min); after that, it was centrifuged at 4000 rpm (10 min, 10 °C). Off-flavors were analyzed in GC/ITMS. The quantitative determination was made in SIM mode using *m/z* 95 and 112 for 2-MIB and GSM, respectively.

### 2.9. Statistical Analysis

Analysis of variance (ANOVA) was carried out with the software XLSTAT (Addinsolf LTD, New York, NY, USA, Version 19.4). Mean comparisons of the effects of treatments were calculated by Fisher’s least significant difference test at *p* ≤ 0.05. Analysis data sets were imported into SIMCA 14 (Umetrics AB, Umea, Sweden) for principal component analysis (PCA) [27].

## 3. Results

### 3.1. Physicochemical Analysis

*M. caephalus* and *L. ramada* fish ranged 1.80 ± 0.50 and 0.75 ± 0.45 kg in weight and 49.80 ± 3.70 and 26.74 ± 1.75 cm in length, respectively. The water content was 47.6 ± 8.4% and 50.7 ± 0.4% (g/100 g ± RSD) in fresh gonads and 13.6 ± 3.4% and 24.8 ± 1.3% (g/100 g ± RSD, FW) in bottarga for *L. ramada* and *M. cephalus*, respectively (Table 1). The ash content was 5.9 ± 7.0% and 4.7 ± 14.7% on fresh gonads and 8.6 ± 4.5% and 7.2 ± 1.6% (g/100 g ± RSD, FW) on bottarga for *L. ramada* and *M. cephalus*, respectively (Table 1). The processing step moderately affected the mineral concentration in the gonads. Dry salting for one hour permitted the diffusion of a moderate amount of salt in the gonads of around 44% of total minerals, acting mainly as a dehydrating agent.

### 3.2. Metals and Metalloids

The ICP–OES analytical method allowed the analysis of 22 metals and metalloids. Calibration curves showed r^2^ values ranging from 0.9967 (Pb) to 0.9997 (Co) and that the limits of quantification of the method were adequate for this study (Table 2).

Among the 22 metals and metalloids studied, only nine could be quantified in all matrices (Table 3). Despite the concentration factor, all metals showed lower concentration in the bottarga; the technological process of salting and removing the salt can affect the content of the metals, decreasing their overall content in the fish. Pb was detected only in low amounts in bottarga samples and was more abundant in *M. cephalus*. Zn was the most abundant both in fresh roe (1.14, and 0.73 µg/g) and bottarga (0.64 and 0.44 µg/g) in *L. ramada* and *M. cephalus*, respectively. Among all other metals, Fe and Al showed higher values in fresh and processed roe, with amounts slightly higher in *M. cephalus*. All metals showed higher levels in *M. cephalus* samples. This fact can be related to their feeding habits in seabed mud (Table 3).

### 3.3. Proteins and Amino Acids

Total protein content ranged from 21.31 to 26.43 g/100 g, showing statistically even values in both species, and this did not change after processing (Table 1).

The analysis carried out on the free amino acids allowed the determination of 11 compounds among the 17 investigated, the most concentrated being glutamine in the fresh roe of both species. Within the same species, there were significant variations in fresh roe vs. bottarga, except for glycine and proline in *L. ramada*, and isoleucine, proline, phenylalanine, and lysine in *M. cephalus*. Glutamine, phenylalanine, and lysine had significantly lower values in the bottarga of *L. ramada*, while there was a lower value of glutamine in those of *M. cephalus*. Proline showed even amounts in all samples (Table 4).

### 3.4. Lipid Fraction

Total lipids were even in the fresh samples of *M. cephalus* and *L. ramada,* accounting for 2.46 and 2.76 g/100 g, respectively; after processing, they increased to 7.46 and 11.31 g/100 g (FW), respectively. The same trend was shown by squalene and cholesterol, which showed higher values in the samples of bottarga. *M. cephalus* showed higher values of cholesterol and squalene than those detected in *L. ramada* (Table 1).

A total of 51 fatty acids were identified in the gonads of both fish. In the fresh gonads of *M. cephalus*, SAFAs represented 20.4%, MUFAs 54.4%, and PUFAs 24.7%. After processing into bottarga, total MUFAs remained unchanged, while SAFAs decreased and PUFAs increased (Table 5 and Table 6).

The same behavior was found in the samples of *L. ramada*. C16:1 mainly represented MUFAs, followed by C18:1Δ9c and C18:1Δ11. C16:0 (between 10% and 15%) and C14:0 (between 2% and 4%) were the SAFAs most represented. Non-negligible concentrations of C15:0 and C18:0 (about 2%) were detected. C17:0 has been identified as a typical chemical indicator of the lipid fraction of *M. cephalus,* according to Pigott et al. (2003) [28], and it was also found in *L. ramada* (Table 5).

PUFAs were represented by DHA (C22:6 n6), EPA (C20:5 n3), and C18:2 conjugated linoleic acid (CLA) isomers. C18:2 and fatty acids with 3 and 4 double bonds increased in bottarga, while EPA and DHA decreased. Short-chain length acids (C14 and C15) decreased both in SAFAs and MUFAs, and an unusual inversion of the oleic–vaccenic ratio was detected in bottarga. Fresh gonads of *L. ramada* showed an amount of SAFAs of 21.9 ± 0.5%, MUFAs of 55.2 ± 1.8%, and PUFAs of 22.4 ± 5.3%, with a behavior similar to *M. cephalus* after processing (Table 5). Unsaturated fatty acids of the ω3 series accounted for 14.01% and 9.95% in fresh roe and 14.24% and 6.44% in bottarga for *L. ramada* and *M. cephalus*, respectively (Table 6). On the other hand, ω6 was 2.17% and 8.56% in fresh samples and 3.28% and 6.07% after processing, respectively (Table 6). The ω3/ω6 ratio was 4.41 and 1.12, respectively, showing a net increase of the ω6 fraction in *L. ramada* bottarga (Table 6).

### 3.5. Geosmine and 2-Methyl Isoborneol

No traces of geosmine and 2-methylisoborneol were found in fresh roe and bottarga in both mullets. The lagoon of Cabras has an elevate ecological resilience to adverse environmental conditions as a potential source of the growth of algae and cyanobacteria. The stability of environmental conditions appears essential in controlling Cyanophyceae development in many cases [29,30,31,32].

### 3.6. Multivariate Analysis

**Figure 1:**
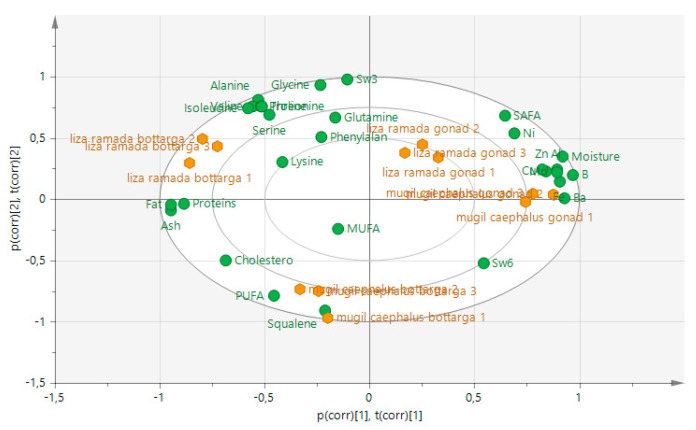
Principal component analysis (PCA) score scatter biplot of fresh gonads and bottarga from the two mullet species.

Principal component analysis (PCA) of all data from the analysis reported showed four clustering groups differentiating within the samples and within the species on the PC1 and PC2 axes (Figure 1).

**Figure 2:**
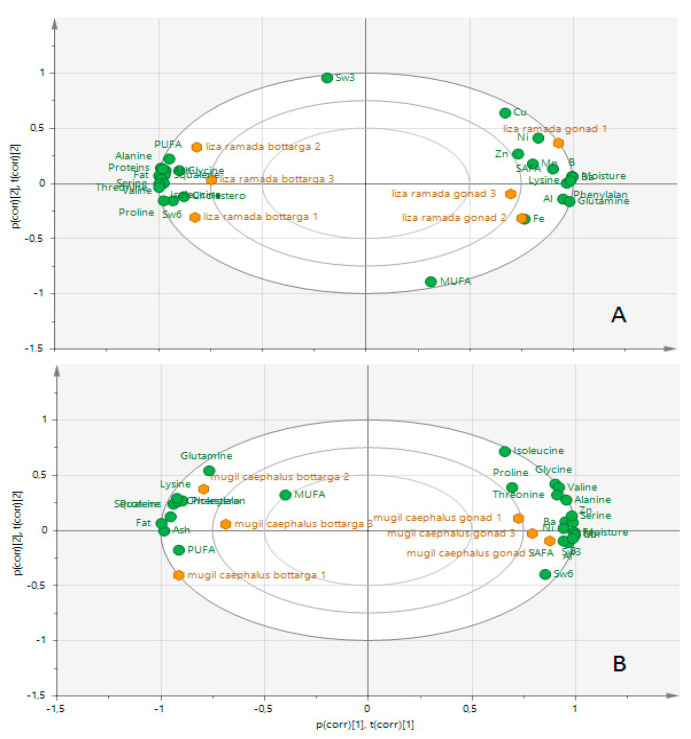
Biplot from PCA analysis of the samples of bottarga vs. fresh roe from *Liza ramada* (**A**), and *Mugil cephalus* (**B**).

A biplot approach was taken to better emphasize the similarities or differences among the groups. *L. ramada* gonads had higher levels than the corresponding bottarga of SAFAs, metals (Cu, Ni, Zn, Mn, Al, Fe, and B), and the amino acids phenylalanine, glutamine, and lysine. In contrast, bottarga had higher values of the other amino acids, total lipid, squalene, cholesterol, PUFAs, and, among these, of ω6 (Figure 2A). *M. caephalus* gonads were rich in ω3, ω6, and SAFAs and the amino acids isoleucine, proline, glycine, valine, alanine, serine, and threonine. At the same time, the corresponding bottarga had higher concentrations of total proteins, total lipid, squalene, cholesterol, PUFAs, and the amino acid glutamine (Figure 2B).

**Figure 3:**
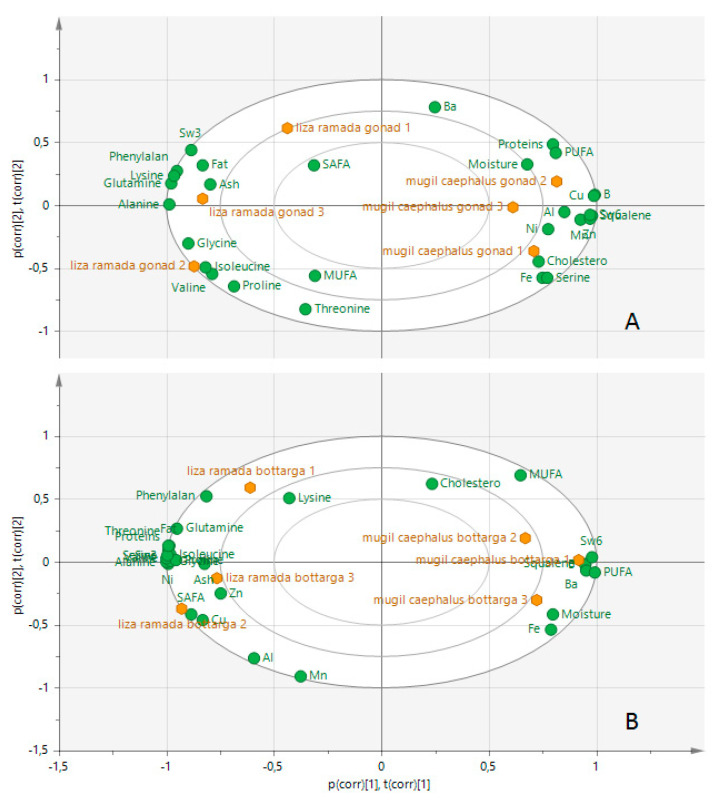
Biplot from PCA analysis of the samples of fresh roe (gonads) (**A**), and bottarga (**B**) from *Liza ramada* vs. *Mugil cephalus*.

Squalene, cholesterol, PUFA ω6, and Fe on the right side of the PC1 axis and isoleucine proline, valine, alanine phenylalanine, glutamine, glycine, and ω3, together with total lipid on the left side of the PC1 axis, allowed us to discriminate *M. caephalus* from *L. ramada* both in bottarga and gonads (Figure 3A,B).

## 4. Discussion

Food processing leads to a final product that is entirely different from the starting raw material, depending on the impact on the biochemical composition. Salting out represents one of the oldest techniques for food, especially fish, preservation and increasing shelf life [33].

In this paper, the entire process of salting has been optimized in terms of time, temperature, and moisture. The rate time/material for the salting step was set at 1 h/200 g, ensuring a moderate dehydrating effect and avoiding an excessive nonhealthy increase of the NaCl content in the bottarga. Celik et al. [16] reported a rate of 2.5 h/200 g and an increase in minerals related to NaCl, like those reported by Barra et al. [12], almost two times the values reported in this paper. The pressing step of one day was adequate to give the product a shape and further reduced moisture. Organoleptic acceptance of a panel of qualified consumers set the ideal moisture content for an appropriate final bottarga consistency at 30%; therefore, the drying days were adapted to the gonad weight.

The data reported on the proximate composition showed similar values with those reported previously by Celik et al. (2012) [16] and Caredda et al. (2018) [17]. Moreover, our data showed similar proximate values for fresh and processed gonads. On the other hand, the values of moisture reported for bottarga were lower than those detected by Lu et al. (1979) [34] and Kalogeropoulos et al. (2008) [35]. This fact can be explained by considering the differences in the salting step carried out under brine (15% NaCl) by Lu et al. and the coverage with beeswax following Greek-style processing by Kalogeropulos et al. Both methods reduced water evaporation, increasing moisture in the bottarga.

Most papers have reported data on samples that were already processed or compared fresh roe versus processed samples from different fish [12,17]. Our samples followed the entire production chain, being samples from the same fish, analyzed before and after processing. This approach allowed a better understanding of the effect of the technological process on biochemical composition.

Total lipids showed higher values in bottarga than in the fresh roe but lower values than those reported by other authors, according to Kalogeropulos et al. [35].

Fatty acid composition of bottarga follows the series MUFA > PUFA > SAFA, according to literature data [6,35,36]; however, the present paper detected a higher level of MUFAs and a lower level of SAFAs. The final ratio of PUFA/SAFA was 40% higher than data from previous studies on roe processed in Sardinia but from different origins.

Moreover, unsaturated fatty acid content was higher than those reported for caviar (4.8%) and *Morone saxatilis* eggs (1.3%) [37,38].

No data are available on the changes in fatty acid and free amino acid composition during processing. Our data showed an increase in PUFAs (C18:2 family) and a decrease in SAFAs (C15:0), with a higher rate of PUFA/SAFA in bottarga then in fresh roes.

Salting can have a substantial effect on protein structure and degradation [39]. Free amino acids are produced by the hydrolysis of proteins, influencing the flavor characteristics of the processed food. The analysis of the free amino acid fraction showed higher values of essential amino acid/total amino acid ratio (EAA/TAA) in bottarga vs. fresh gonads. Among the 11 free amino acids detected, the analysis showed a general increase of all compounds in bottarga except for glutamine, which decreased in all samples.

The data of metals and metalloids showed that the salting step significantly affected their concentration. Decreasing rates ranged from 35.1% (Cu) to 77.0% (Al), assuming a possible removal effect associated with water extraction by the external salt. Amir et al. (2018) reported the performance of acidic and alkaline solutions and biological extracts in reducing heavy metal content from spinach after dip washing [40].

Multivariate analysis is a powerful tool for sample discrimination, and it has been used to differentiate among bottarga of different origins [12]. In this paper, PCA allowed us to evaluate the significance of the biochemical changes among fresh roe and bottarga and between *L. ramada* and *M. cephalus* composition.

The biochemical analysis allowed us to perform a nutritional evaluation of the bottarga of the two fish species. Fish roe consumption is diffused in the world; however, different considerations on the nonhealthy effect of some chemical components have been made.

Cholesterol quantitation showed different results, varying from 290 to 590 mg/100 g [41]. Considering the levels of cholesterol found in this paper and an FDI of bottarga around 10 g, the overall intake could reach values of cholesterol between 47.5 and 72.2 mg, which is almost three times lower than an egg of 50 g (180 mg of cholesterol). *L. ramada* processed roe showed levels of cholesterol lower than those reported by Rosa et al. [6] and similar to those reported by Kalogeropoulos [35] and Barra [12].

The high levels of squalene and ω3 fatty acid will provide a relevant health profile. A high PUFA content is related to a high nutritional value of food [28,42]; moreover, recent research pointed out the health benefits of ω3 as the precursor of bioactive epoxides. These ω3 endocannabinoid epoxides interact with THC receptors, playing crucial roles in modulating neuroinflammation and cerebrovascular disease [43].

Amino acids are essential components of the human diet for body health; glycine, serine, alanine, proline, and other amino acids are involved in tissue regrowth and healing. Glutamine is necessary for cell proliferation. Essential amino acids are not produced in human bodies but are assimilated from food. Among the eight amino acids generally regarded as essential for humans, in our samples, we found phenylalanine, valine, threonine, isoleucine, leucine, and lysine.

The two species of mullets result in good sources of amino acids, with values according to WHO/FAO/UNU indications for human nutrition [44].

## 5. Conclusions

Bottarga represents a highly priced delicacy, and its consumption is usually limited to appetizers, sashimi, or as a sauce for pasta.

This paper has reported the influence of industrial processing on the biochemical composition of fresh roe from two mugilid species, *Liza ramada* and *Mugil cephalus*. Eight different chemical analyses were performed for the characterization of the bottarga produced from the fresh roe. The processing step affected the quality and composition of the bottarga, which showed an attractive nutritional value due to its lipidic profile in both fatty acid and squalene, low levels of cholesterol, and an increased EAA/TAA ratio. PCA analysis showed clear discrimination among the two species in both fresh roe and bottarga. *L. ramada* showed a better ratio of ω3/ω6 but lower values of squalene and cholesterol. Finally, the paper reported, for the first time, a comprehensive evaluation of metal and metalloid composition.

In addition to nutritional benefits for the consumer, the findings of the paper also have a significant impact on the producers of processed roe. The leading enterprises have a codified and, sometimes, patented processing method to obtain homogeneous products with unchanging organoleptic characteristics. Moreover, small and local producers make use of empirical and traditional methodologies, leading to different products among the same batch and between batches. Thus, the process set in this paper may represent an easy-to-use and reliable tool to obtain a final bottarga, with both a reproducible biochemical composition and organoleptic characteristics.

## Figures and Tables

**Table 1:** Chemical–physical analysis, total protein, total lipid (g/100 g, FW), squalene, and cholesterol (mg/100 g, FW) of fresh roe and bottarga from *M. cephalus* and *L. ramada*.

Species	Sample	Humidity (%) g/100 g FW	Ash g/100 g FW	Total Protein g/100 g FW	Total Lipid g/100 g FW	Squalene mg/100 g	Cholesterol mg/100 g
*L. ramada*	fresh	47.6 ^a^	5.9 ^a^	24.5 ^a^	2.7 ^a^	11.3 ^a^	474.7 ^a^
	bottarga	13.6 ^b^	8.6 ^b^	24.7 ^a^	11.3 ^b^	24.6 ^b^	621.0 ^b^
*M. cephalus*	fresh	50.7 ^a^	4.7 ^a^	26.4 ^a^	2.5 ^a^	49.2 ^c^	648.9 ^b^
	bottarga	24.9 ^c^	7.2 ^b^	21.3 ^a^	7.5 ^c^	68.2 ^d^	722.0 ^c^

In each column, means followed by a common letter are not significantly different by Fisher’s least significant difference (LSD) procedure, *p* ≤ 0.05.

**Table 2:** Metal method parameters, limit of quantification (LOQ), correlation coefficient, and quantification wavelength (λ).

		LOQ		
Metal	λ	(µg/kg)	Linear Regression Equation	r^2^
Al	396.15	0.05	y = 670.89x − 45.48	0.9978
As	193.69	0.05	y = 79.38x − 10.14	0.9991
B	249.67	0.025	y = 5151.33x + 789.8	0.9995
Ba	455.40	0.05	y = 8035x − 289.9	0.9993
Be	313.10	0.001	y = 259,128x − 6848	0.9985
Cd	214.43	0.005	y = 4804x − 3.55	0.9995
Co	238.89	0.01	y = 6622.7x + 58.6	0.9997
Cr	205.56	0.025	y = 9908.7x + 97.7	0.9994
Cu	324.75	0.01	y = 17,117.2x − 95.93	0.9996
Fe	238.20	0.10	y = 3059.9x + 80.8	0.9992
Hg	194.16	0.10	y = 392.2x + 11	0.9994
Mn	257.61	0.005	y = 70,780.6x − 8029	0.9990
Mo	204.59	0.05	y = 604.4x − 5.5	0.9988
Ni	231.60	0.025	y = 1642.2x − 22.3	0.9986
Pb	220.35	0.10	y = 297.3x − 19.4	0.9967
Sb	206.83	0.10	y = 49.7x − 5.8	0.9981
Sn	189.92	0.10	y = 74.3x − 4.9	0.9986
Sr	407.71	0.01	y = 829,945.3x + 17943	0.9984
Te	214.28	0.50	y = 106.6x − 10.1	0.9972
Ti	336.12	0.10	y = 14,275x + 521.8	0.9976
V	292.40	0.005	y = 13,753.2x + 231.2	0.9996
Zn	213.85	0.10	y = 4785.9x − 316	0.9986

**Table 3:** Heavy metal composition (µg/kg FW) of fresh roe and bottarga of *M. cephalus* and *L. ramada* collected from Sardinia lagoon.

		*Liza ramada*		*Mugil cephalus*		
Metal	Fresh	Bottarga	Decrease	Fresh	Bottarga	Decrease
	μg/kg {\displaystyle \sigma}		%	μg/kg {\displaystyle \sigma}		% {\displaystyle \sigma}
Al	88.2 ^a^	26.2 ^b^	70.3	121.4 ^a^	27.9 ^b^	77.0
As	<LOQ	<LOQ		<LOQ	<LOQ	
B	47.1 ^a^	14.9 ^b^	68.4	67.5 ^a^	19.3 ^b^	71.4
Ba	5.4 ^a^	1.8 ^b^	66.7	4.8 ^a^	1.8 ^b^	62.5
Be	<LOQ	<LOQ		<LOQ	<LOQ	
Cd	<LOQ	<LOQ		<LOQ	<LOQ	
Co	<LOQ	<LOQ		<LOQ	<LOQ	
Cr	<LOQ	<LOQ		<LOQ	<LOQ	
Cu	17.3 ^c^	10.9 ^c^	37.0	43.6 ^a^	28.3 ^b^	35.1
Fe	206.9 ^b^	98.2	52.5	358.4 ^a^	159.3 ^b^	55.6
Hg	<LOQ	<LOQ		<LOQ	<LOQ	
Mn	11.4 ^a^	4.6 ^c^	59.6	18.4 ^a^	7.2 ^b^	60.9
Mo	<LOQ	<LOQ		<LOQ	<LOQ	
Ni	4.1 ^b^	2.6 ^c^	36.6	7.2 ^a^	4.6 ^b^	36.1
Pb	<LOQ	8.4 ^b^		<LOQ	14.1 ^a^	
Sb	<LOQ	<LOQ		<LOQ	<LOQ	
Sn	<LOQ	<LOQ		<LOQ	<LOQ	
Sr	5.3 ^c^	1.9 ^b^	64.2	7.4 ^a^	2.9 ^b^	60.8
Te	<LOQ	<LOQ		<LOQ	<LOQ	
Ti	<LOQ	<LOQ		<LOQ	<LOQ	
V	<LOQ	<LOQ		<LOQ	<LOQ	
Zn	729.2 ^b^	441.0 ^c^	39.5	1143.5 ^a^	635.9 ^b^	44.4

In each row, means followed by a common letter are not significantly different by Fisher’s least significant difference (LSD) procedure, *p* ≤ 0.05.

**Table 4:** Free amino acid composition (% ± RSD) of fresh roe and bottarga from *L. ramada* and *M. cephalus* samples.

Aminoacid	*Liza ramada*		*Mugil cephalus*	
	Fresh	Bottarga	Fresh	Bottarga
Alanine	8.8 ^a*^	13.1 ^b^	7.3 ^a^	11.4 ^b^
Glycine	7.2 ^a^	8.4 ^a^	6.6 ^a^	12.8 ^b^
Valine ^e^	6.3 ^a^	11.2 ^b^	5.6 ^a^	9.4 ^b^
Isoleucine ^e^	5.1 ^a^	8.3 ^b^	4.3 ^a^	6.6 ^a^
Proline	5.2 ^a^	7.8 ^a^	4.7 ^a^	5.1 ^a^
Serine	7.4 ^a^	16.3 ^b^	9.3 ^a^	14.7 ^b^
Threonine ^e^	6.4 ^a^	11.7 ^b^	6.3 ^a^	10.2 ^b^
Glutamine	25.6 ^a^	13.6 ^b^	20.1 ^a^	12.3 ^b^
Phenylalanine ^e^	9.1 ^a^	5.2 ^b^	7.5 ^a^	6.3 ^a^
Lysine ^e^	6.6 ^a^	4.0 ^b^	6.2 ^a^	7.1 ^a^
Tyrosine ^e^		0.2 ^a^		1.2 ^b^
TAA	87.70 ± 3.31	99.80 ± 0.02	47.48 ± 0.54	38.78 ± 3.64
EAA	33.47 ± 2.35	40.46 ± 1.01	17.80 ± 0.11	17.11 ± 2.30
EAA/TAA%	38.17 ± 0.97	40.54 ± 0.99	37.24 ± 0.66	44.14 ± 3.00

* In each raw grouping, means followed by a common letter are not significantly different by Fisher’s least significant difference (LSD) procedure, *p* ≤ 0.05. ^e^ essential amino acids.

**Table 5:** Fatty acid composition (%) of fresh roe and bottarga of *M. cephalus* and *L. ramada* collected from Sardinia lagoon.

Fatty Acid	*Liza ramada*		*Mugil cephalus*	
	Fresh	Bottarga	Fresh	Bottarga
anteiso C14	0.03 ^a*^	0.04 ^a^	0.03 ^a^	0.03 ^a^
C14:0	3.55 ^b^	1.99 ^a^	2.73 ^b^	1.60 ^a^
anteiso C15:0	0.18 ^b^	0.08 ^a^	0.14 ^b^	0.09 ^a^
C15:0 + isomer	2.04 ^c^	1.37 ^a^	1.64 ^b^	1.50 ^b^
anteiso C16	1.28 ^b^	1.93 ^c^	1.83 ^c^	0.04 ^a^
C16:0	12.60 ^b^	12.84 ^b^	9.60 ^a^	9.16 ^a^
C17:0	0.42 ^b^	0.74 ^c^	0.25 ^a^	0.57 ^b^
C18:0	1.76 ^b^	1.70 ^b^	1.01 ^a^	1.46 ^b^
anteiso C19	0.05	0.04 ^a^	0.06 ^a^	0.05
C20:0	0.09 ^a^	0.14 ^b^		
C14:1 + isomer	0.11 ^a^	0.03 ^a^	0.38 ^b^	0.08 ^a^
C15:1 + isomer	0.72 ^b^	0.33 ^a^	0.77 ^b^	0.51 ^a^
C16:1 + isomer	30.45 ^b^	27.18 ^a^	30.09 ^b^	24.85 ^a^
C16:2	0.32 ^a^	0.42 ^a^	0.40 ^a^	
C16:1 n9 7 methyl	0.15 ^a^	0.22 ^b^	0.12 ^a^	0.13 ^a^
C17:1	1.68 ^a^	2.81 ^b^	1.57 ^a^	3.00 ^b^
C18:1c	16.32 ^b^	12.74 ^a^	16.06 ^b^	13.01 ^a^
C18:1 Δ11	5.30 ^a^	10.33 ^b^	5.29 ^a^	11.42 ^b^
C20:1	0.13 ^a^		0.13 ^a^	0.56 ^b^
C20:1n7	0.13 ^a^	0.32 ^b^	0.22 ^b^	0.10 ^a^
C20:1n4	0.23 ^b^	0.17 ^a^	0.26 ^b^	
C17:2	1.36 ^b^	0.80 ^a^	1.18 ^b^	2.68 ^c^
C18:2	0.55 ^a^	2.65 ^c^		1.75 ^b^
C18:2n6t	0.17 ^a^	0.42 ^b^	1.44 ^c^	1.76 ^c^
C18:2 n6c	1.17 ^c^	0.75 ^b^	0.20 ^a^	1.34 ^c^
C18:2	0.66 ^b^	0.52 ^b^	1.27 ^c^	0.17 ^a^
C18:3	0.22 ^a^	0.32 ^a^	0.70 ^b^	0.26 ^a^
γ C18:3n6	0.34 ^a^	0.47 ^ab^	0.20 ^a^	0.53 ^b^
αC18:3	1.16 ^c^	0.81 ^bc^	0.43 ^a^	0.55 ^b^
unknow	1.98 ^b^	1.74 ^a^	2.03 ^b^	
C18:4n3	1.20 ^b^	0.60 ^a^	3.94 ^c^	4.28 ^c^
CLA C18:2	1.21 ^b^	0.69 ^a^	1.11 ^b^	1.61 ^b^
CLA C18:2	0.80 ^a^	2.59 ^c^	1.71 ^b^	2.57 ^c^
C20:2 + isomer	0.17 ^a^	0.23 ^b^	1.56 ^b^	0.15 ^a^
C20:3	0.21 ^a^	2.36 ^b^	0.14 ^a^	0.25 ^a^
C20:4	0.44 ^a^	1.06 ^b^	0.45 ^a^	1.35 ^b^
C20:3n3 (11-14-17)	0.07 ^a^	0.10 ^a^	0.08 ^a^	0.13 ^a^
C20:4n3	0.74 ^a^	2.77 ^b^	0.90 ^a^	0.95 ^a^
C20:5 EPA	3.00 ^b^	1.41 ^a^	3.71 ^b^	2.19 ^ab^
C20:3n9	0.11 ^a^		0.07 ^a^	
C21:4	0.36 ^b^	0.17 ^a^	0.42 ^b^	0.12 ^a^
C21:5n3	0.36 ^b^	0.15 ^a^	0.23 ^a^	0.31 ^ab^
C22:5	1.83 ^a^	2.12 ^b^	1.92 ^ab^	
C22:6 DHA	4.37 ^b^	2.00 ^a^	3.50 ^b^	

* In each raw grouping, means followed by a common letter are not significantly different by Fisher’s least significant difference (LSD) procedure, *p* ≤ 0.05.

**Table 6:** Fatty acid composition (%) of fresh roe and bottarga of *M. cephalus* and *L. ramada* collected from Sardinia lagoon.

	*Liza ramada*		*Mugil cephalus*	
	Fresh	Bottarga	Fresh	Bottarga
Σsaturated	21.98 ^b^	20.84 ^b^	17.34 ^ab^	14.58 ^a^
Σmonounsaturated	55.55 ^a^	54.44 ^a^	54.80 ^a^	56.07 ^a^
Σpolyunsaturated	22.47 ^a^	24.72 ^ab^	26.21 ^bc^	29.36 ^c^
PUFA/SAFA	1.02 ^a^	1.59	1.19 ^a^	2.03
Σω3 (%)	14.01 ^c^	14.24 ^c^	9.95 ^b^	6.44 ^a^
Σω6 (%)	2.17 ^a^	3.28 ^a^	8.56 ^c^	6.07 ^b^
ω3/ω6	6.44 ^b^	4.41 ^b^	1.17 ^a^	1.12 ^a^

In each raw grouping, means followed by a common letter are not significantly different by Fisher’s least significant difference (LSD) procedure, *p* ≤ 0.05.

## References

[1] Crosetti D., Blaber S.J.M. (2016). Biology, Ecology and Culture of Grey Mullets (Mugilidae).

[2] Saleh M.A. (2006). Cultured Aquatic Species Information Programme.

[3] Saleh M., A. Lovatelli A., Holthus P.F. (2008). Capture-based aquaculture of mullets in Egypt. Capture-Based Aquaculture, Technical Paper.

[4] Thomson J.M., Quero J.C., Hureau J.C., Post C.A., Saldanha L. (1990). Mugelidae. Check List of the Fishes of the Eastern Tropical Atlantic.

[5] Bledsoe G.E., Bledsoe C.D., Rasco B. (2003). Caviars and fish roe products. Crit. Rev. Food Sci. Nutr..

[6] Rosa A., Atzeri A., Deiana M., Melis M.P., Ancani A., Loru D., Cabboi B., Dessì M.A. (2014). Mullet bottarga as functional ingredient. Aliment. Funz..

[7] Brownlie I. (1998). The Rule of Law in International Affairs: International Law at the Fiftieth Anniversary of the United Nations.

[8] Commission Implementing Regulation (EU) (2017). EU 2017/1925 of 12 October 2017, Amending Annex I to Counci Regulation (EEC) No 2658/87 on the Tariff and Statistical Nomenclature and on the Common Customs Tariff.

[9] Food and Forestry Policies (MIPAAF) (2018). Update of the National List of Traditional Agri-Food Products Pursuant to Article 12, Paragraph 1, of the Law of 12 December 2016, n. 238.

[10] Angioni A., Cau A., Addis P. (2014). Gas chromatographic mass spectrometry determination of geosmin and 2-methylisoborneol off-Flavor in Mugil cephalus roe. Food Anal. Methods.

[11] FAO Fisheries and Aquaculture Department Rome FAO Major Fishing Areas. http://www.fao.org/fishery/area/search/en.

[12] Barra A., Garau V.L., Dessi S., Sarais G., Cereti E., Arlorio M., Coisson J.D., Cabras P. (2008). Chemical characterization and DNA tracking of Sardinian botargo by Mugil cephalus from different geographical origins. J. Agric. Food Chem..

[13] Locci E., Piras C., Mereu S., Cesare Marincola F., Scano P. (2011). 1H NMR Metabolite fingerprint and pattern recognition of Mullet (Mugil cephalus) Bottarga. J. Agric. Food Chem..

[14] Scano P., Rosa A., Locci E., Dessì M.A., Lai A. (2009). NMR study of the lipid profile of mullet raw roe and bottarga. Eur. J. Lipid Sci. Technol..

[15] Scano P., Rosa A., Locci E., Manzo G., Dessì M.A. (2012). Modifications of the 1H NMR metabolite profile of processed mullet (Mugil cephalus) roes under different storage conditions. Magn. Reson. Chem..

[16] Çelik U., Altınelataman C., Dinçer T., Acarlı D. (2012). Comparison of Fresh and Dried Flathead Grey Mullet (*Mugil cephalus*, Linnaeus 1758) Caviar by means of Proximate Composition and Quality Changes during Refrigerated Storage at 4 ± 2 °C. Turk. J. Fish. Aquat. Sci..

[17] Caredda M., Addis M., Pes M., Fois N., Sanna G., Piredda G., Sanna G. (2008). Physico-chemical, colorimetric, rheological parameters and chemometric discrimination of the origin of Mugil cephalus’ roes during the manufacturing process of bottarga. Food Res. Int..

[18] Murenu M., Olita A., Sabatini A., Follesa M.C., Cau A. (2004). Dystrophy effects on the Liza ramada (Pisces, Mugilidae) population in the Cabras lagoon (Central-Western Sardinia). Chem. Ecol..

[19] Cataudella S., Crosetti D., Massa F. (2015). Mediterranean coastal lagoons: Sustainable management and interactions among aquaculture, capture fisheries and the environments. Int. Stud. Rev..

[20] Padedda B.M., Pulina S., Magni P., Sechia N., Luglie A. (2012). Phytoplankton dynamics in relation to environmental changes in a phytoplankton-dominated Mediterranean lagoon (Cabras Lagoon, Italy). Adv. Oceanogr. Limnol..

[21] World Organization of Animal Health Welfare Aspects of Stunning and Killing of Farmed Fish for Human Consumption. https://www.oie.int/en/standard-setting/aquatic-code/access-online/.

[22] Corrias F., Atzei A., Addis P., Secci M., Russo M., Angioni A. (2020). Integrated environmental evaluation of heavy metals and metalloids bioaccumulation in invertebrates and seaweeds from different marine coastal areas of Sardinia, Mediterranean Sea. Environ. Pollut..

[23] Beach L.M. Determination of As, Sb and Se in Difficult Environmental Samples by Hydride Generation. https://www.agilent.com/cs/library/applications/aa105.pdf.

[24] Evans S.J., Johnson M.S., Leah R.T. Determination of Mercury in Fish Tissue, a Rapid, Automated Technique for Routine Analysis. https://www.agilent.com/cs/library/applications/AA060.pdf.

[25] Wenzl T., Haedrich J., Schaechtele A., Robouch P., Stroka J. (2016). Guidance Document on the Estimation of LOD and LOQ for Measurements in the Field of Contaminants in Feed and Food. EUR 28099.

[26] Srigley C.T., Mossoba M.M., Spizzirri U.G., Cirillo G. (2017). Current Analytical Techniques for Food Lipids. Food Safety: Innovative Analytical Tools for Safety Assessment.

[27] Atherton H.J., Bailey N.J., Zhang W., Taylor J., Major H., Shockcor J., Clarke K., Griffin J.L. (2006). A combined 1H NMR spectroscopy and mass spectrometry-based metabolomic study of the PPAR-null mutant mouse defines profound systemic changes in metabolism linked to the metabolic syndrome. Physiol. Genomics.

[28] Pigott G.M., Tuker B.W. (2003). Fish oils. Encyclopaedia of Food Sciences and Nutrition.

[29] Chomérat N., Garnier R., Bertrand C., Cazaubon A. (2007). Seasonal succession of cyanoprokaryotes in a hyperetrophic oligo-mesohaline lagoon from the South of France. Estuar. Coast. Shelf Sci..

[30] Pulina S., Padedda B.M. (2011). The dominance of cyanobacteria in Mediterranean hypereutrophic lagoons: A case study of Cabras Lagoon (Sardinia, Italy). Sci. Mar..

[31] Scheffer M., Rinaldi S., Gragnani A., Mur L.R., Van Nes E.H. (2007). On the dominance of filamentous cyanobacteria in shallow. Turbid Lakes. Environ..

[32] Sorokin Y.I., Sorokin P.Y., Gnes A. (1996). Structure and functioning of antropogenically transformed Comacchio lagoon ecosystem (Ferrara, Italy). Mar. Ecol. Prog. Ser..

[33] Hafez N.E., Awad A.M., Ibrahim S.M., Mohamed H.R., El-Lahamy A.A. (2019). Effect of Salting Process on Fish Quality. Nutr. Food Process..

[34] Lu J.Y., Ma Y.M., Williams C., Chung R.A. (1979). Fatty and amino acid composition of salted mullet roe. J. Food Sci..

[35] Kalogeropoulos N., Nomikos T., Chiou A., Fragopoulou E., Antonopoulou S. (2008). Chemical composition of Greek Avgotaracho prepared from mullet (Mugil cephalus): Nutritional and health benefits. J. Agric. Food Chem..

[36] Scano P., Rosa A., Cesare Marincola F., Locci E., Melis M.P., Dessì M.A., Lai A. (2008). 13C NMR, GC and HPLC characterization of lipid components of the salted and dried mullet (Mugil cephalus) roe “bottarga”. Chem. Phys. Lipids.

[37] Gallagher M.L., McLeod S.H., Rulifson R. (1989). Seasonal variations in fatty acids of striped bass Morone saxatilis. J. World Aquac. Soc..

[38] Wirth M., Kirschbaum F., Gessner J., Williot P., Patriche N., Billard R. (2002). Fatty acid composition in sturgeon caviar from different species: Comparing wild and farmed origins. Int. Rev. Hydrobiol..

[39] Porarinsdottir K.A. (2010). The Influence of Salting Procedures on the Characteristics of Heavy Salted Cod. Ph.D. Thesis.

[40] Amir R.M., Randhawa M.A., Sajid M.W., Nadeem M., Ahmad A., Watto F.M. (2019). Evaluation of various soaking agents as a novel tool for heavy metal residues mitigation from spinach. Food Sci. Technol..

[41] Oluk C.A., Karaca O.B., Grumezescu M.A. (2016). Functional food ingredients and nutraceuticals, milk proteins as nutraceuticals nanoscience and food industry. Nutraceuticals, Nanotechnology in the Agri-Food Industry.

[42] Shirai N., Higuchi T., Suzuki H. (2006). Analysis of lipid classes and the fatty acid composition of the salted fish roe food products, Ikura, Tarako, Tobiko, and Kazunoko. Food Chem..

[43] McDougle D.R., Watson J.E., Abdeen A.A., Adili R., Caputo M.P., Krapf J.E., Johnson R.W., Kilian K.A., Holinstate M., Das A. (2017). Anti-inflammatory ω-3 endocannabinoid epoxides. PNAS Early Ed..

[44] WHO (2002). Protein and Amino Acid Requirements in Human Nutrition. WHO Technical Report Series 935. Joint FAO/WHO/UNU Expert Consultation on Protein and Amino Acid Requirements in Human Nutrition.

